# Mellitate: A multivalent anion with extreme charge density causes rapid aggregation and misfolding of wild type lysozyme at neutral pH

**DOI:** 10.1371/journal.pone.0187328

**Published:** 2017-10-30

**Authors:** Grzegorz Ścibisz, Robert Dec, Wojciech Dzwolak

**Affiliations:** Faculty of Chemistry, Biological and Chemical Research Centre, University of Warsaw, Warsaw, Poland; Russian Academy of Medical Sciences, RUSSIAN FEDERATION

## Abstract

Due to its symmetric structure and abundance of carboxyl groups, mellitic acid (MA–benzenehexacarboxylic acid) has an uncommon capacity to form highly ordered molecular networks. Dissolved in water, MA dissociates to yield various mellitate anions with pronounced tendencies to form complexes with cations including protonated amines. Deprotonation of MA at physiological pH produces anions with high charge densities (MA^5-^ and MA^6-^) whose influence on co-dissolved proteins has not been thoroughly studied. As electrostatic attraction between highly symmetric MA^6-^ anions and positively charged low-symmetry globular proteins could lead to interesting self-assembly patterns we have chosen hen egg white lysozyme (HEWL), a basic stably folded globular protein as a cationic partner for mellitate anions to form such hypothetical nanostructures. Indeed, mixing of neutral HEWL and MA solutions does result in precipitation of electrostatic complexes with the stoichiometry dependent on pH. We have studied the self-assembly of HEWL-MA structures using vibrational spectroscopy (infrared absorption and Raman scattering), circular dichroism (CD), atomic force microscopy (AFM). Possible HEWL-MA^6-^ molecular docking scenarios were analyzed using computational tools. Our results indicate that even at equimolar ratios (in respect to HEWL), MA^5-^ and MA^6-^ anions are capable of inducing misfolding and aggregation of the protein upon mild heating which results in non-native intermolecular beta-sheet appearing in the amide I’ region of the corresponding infrared spectra. The association process leads to aggregates with compacted morphologies entrapping mellitate anions. The capacity of extremely diluted mellitate anions (i.e. at sub-millimolar concentration range) to trigger aggregation of proteins is discussed in the context of mechanisms of misfolding.

## Introduction

Mellitic acid is a remarkably stable intermediate product of oxidation of polycyclic aromatic hydrocarbons and graphite. MA has several interesting molecular and physicochemical properties barely explored in the context of interactions with biomolecular systems. The stability and non-volatile character of MA have made it one of the key reporter molecules for organic matter in astrochemistry [1–3]. In aqueous solutions, MA undergoes gradual dissociation with the corresponding pK_a_ values spread in the range between approximately 1 and 7 [4–6]. The high symmetry of MA molecule, the chemical uniformity of carboxyl groups with their capacity to form, depending on pH, hydrogen bonds and salt bridges, underlie the significant potential of MA and its derivatives as building blocks for self-assembled molecular networks [7–12] and as substrates for chemical cross-linking and polymerization [13–14]. Various mellitate anions exhibit pronounced propensity to form complexes with cationic species including transition metals (e.g. [15–16]) and protonated amines [7, 10]. However, there are no systematic studies on such complexes when the cationic component is a basic protein. Several groups have employed MA either for covalent modification of enzymes aimed at achieving a higher degree of thermal stability [17], or to modulate oxygen affinity of hemoglobin [18]. Elsewhere, it was suggested that the vast spread of pK_a_ values of MA and its large buffer capacity would make it advantageous as an ingredient of media used for *in vitro* growth of bacterial cultures [19]. Whitesides et al. showed that several proteins modified by covalent attachment of mellitic acid form charge ladders visible in capillary electrophoresis [20].

At neutral pH, an electrostatic attraction between highly symmetric MA^6-^ anions and positively charged low-symmetry globular proteins could lead to self-assembly patterns distinct from the molecular networks of MA and symmetric organic cations studied so far. The initial motivation of our study was to verify this possibility by matching mellitate with a basic stably folded globular protein. For this task hen egg white lysozyme (HEWL) was selected. While we have observed formation of electrostatic complexes of lysozyme and MA which initially retained the native structure of the protein, we noticed that HEWL molecules involved in these assemblies have a strong tendency to misfold and aggregate, even at very low concentrations of MA. We have discussed our results in the context of mechanisms of protein aggregation.

## Materials and methods

### Samples

HEWL, MA, Thioflavin T (ThT) and other chemicals were purchased from Sigma, USA. Deuterated compounds: D_2_O, NaOD, and DCl were purchased from ARMAR Chemicals, Switzerland. As Fourier-Transform Infrared (FT-IR) spectroscopy in the region of amide I/I’ vibrations was used to probe HEWL conformation, in most cases H_2_O was replaced with D_2_O to prevent spectral overlap of this band with bending vibrations of H_2_O molecules. Unless otherwise stated in figure captions, the following protocol of preparation of aggregates was employed. Fresh 3 wt. % solutions of HEWL and MA in D_2_O were pD-adjusted with diluted NaOD or DCl to specified values, typically, in the range of 7.4–7.6 where MA is effectively deprotonated and MA^5-^ and MA^6-^ anions are abundant; ‘pD’ corresponds to uncorrected pH-meter readout. Precipitating aggregates were subjected to prolonged incubation at specified temperatures (typically at 65 ^o^C) using Eppendorf Thermomixer Comfort accessory. Subsequently, aggregates were subjected to spectroscopic and microscopic measurements.

### FT-IR spectra

For acquisition of FT-IR spectra, 32 interferograms of 2 cm^-1^ nominal resolution were co-added. The spectra were acquired using a CaF_2_ transmission cell and 0.025 mm Teflon spacers on Nicolet iS50 FT-IR spectrometer (Thermo, USA) equipped with a DTGS detector. During measurements, the spectrometer’s sample chamber was continuously purged with dry air. Spectra were collected under ambient temperature conditions. Solvent spectra subtraction, baseline correction and intensity normalization were performed using GRAMS software (from Thermo, USA).

### AFM

Collected samples of aggregates were initially diluted 250 times with H_2_O (pH 7). A 10 μl droplet of aggregate’s suspension was swiftly deposited onto freshly cleaved mica and left to dry for 24 hours. AFM ScanAsyst (R) tapping-mode measurements were carried out using Dimension Icon atomic force microscope (Bruker, USA) and ScanAsyst-AIR sensors, res. frequency 70 kHz (Bruker, USA).

### CD

Samples for CD measurements were prepared by mixing equal volumes of HEWL dissolved in D_2_O, pD 7,4 at 2∙10^−3^ wt. % concentration and sodium mellitate solution in D_2_O of the same molar concentration and pD as the HEWL solution. A quartz cuvette with a 10-mm-optical pathway placed in a PC-controlled Peltier unit was used to collect spectra on Jasco J-815 S spectropolarimeter (Jasco, Japan). Each spectrum was obtained through the accumulation of five independent scans at selected temperature. The temperature in the cell was increased through the Peltier module in a stepwise manner (typically at the rate of 10°C / 15 min). After reaching the highest temperature (85 ^o^C) cell was gradually cooled down to 25 ^o^C within 30 minutes and the final CD spectrum was collected.

### Raman measurements

Raman spectra were collected on DXR Raman Microscope from Thermo Scientific equipped with a 532 nm laser operating at 2 mW power output. For each spectrum 5 independent scans were averaged yielding final spectral resolution in the range between 5.5 and 8.3 cm^-1^. All spectra were corrected for residual background fluorescence using a 4^th^ order polynomial function.

### Fluorescence of thioflavin T (ThT), static light scattering

Aggregates formed through the mixing of 3 wt. % stock solutions of HEWL and MA at specified pD values and at various MA:HEWL molar ratios were incubated for various periods of time and subsequently diluted to 0.01 (for ThT fluorescence measurements) or 0.06 (for light scattering measurements) protein wt. % concentration with H_2_O. For ThT fluorescence emission measurements, the dye was added to the final concentration of 25 μM; ThT fluorescence was excited at 440 nm. After a brief incubation, fluorescence measurements were carried out using 10 mm quartz cuvettes on an AMINCO Bowman Series 2 luminescence spectrometer. Measurements of static light scattering at 532 nm were conducted using the same cuvette and spectrometer setup but in the absence of ThT.

### Simulation of docking interactions and prediction of aggregation-prone fragments in HEWL

For the assessment of HEWL-mellitate docking interactions, a 3D structure of HEWL from Protein Data Bank (PDB entry 2VB1) was used. Water and ligand molecules were deleted using Chimera software. Structure of MA^5-^ ion was obtained from ZINC database (ZINC23329815 record). Structure of MA^6-^ was obtained from CDS database (mellite crystallographic structure, SOGGEA01 record [21], CCDC number 1052513). Dockings were performed using Autodock 4.2.6 software [22]. Autogrid 4 application was run using default parameters. Autodock 4 application was run using default parameters of Genetic Algorithm (Maximum Numbers of evaluations set as ‘long’ (25∙10^6^); number of GA runs was set to 100). Among the resulting structures of HEWL-mellitate complexes, several recurring docking modes (referred to as ‘clusters’) were identified. For each cluster, mean interaction energy and respective standard deviations (SD) were calculated. Aggregation propensities of HEWL sequence fragments were determined by Tango algorithm [23–25]. Sequences with AGG score larger than 1 were considered as aggregation-prone regions.

## Results and discussion

**Fig 1:**
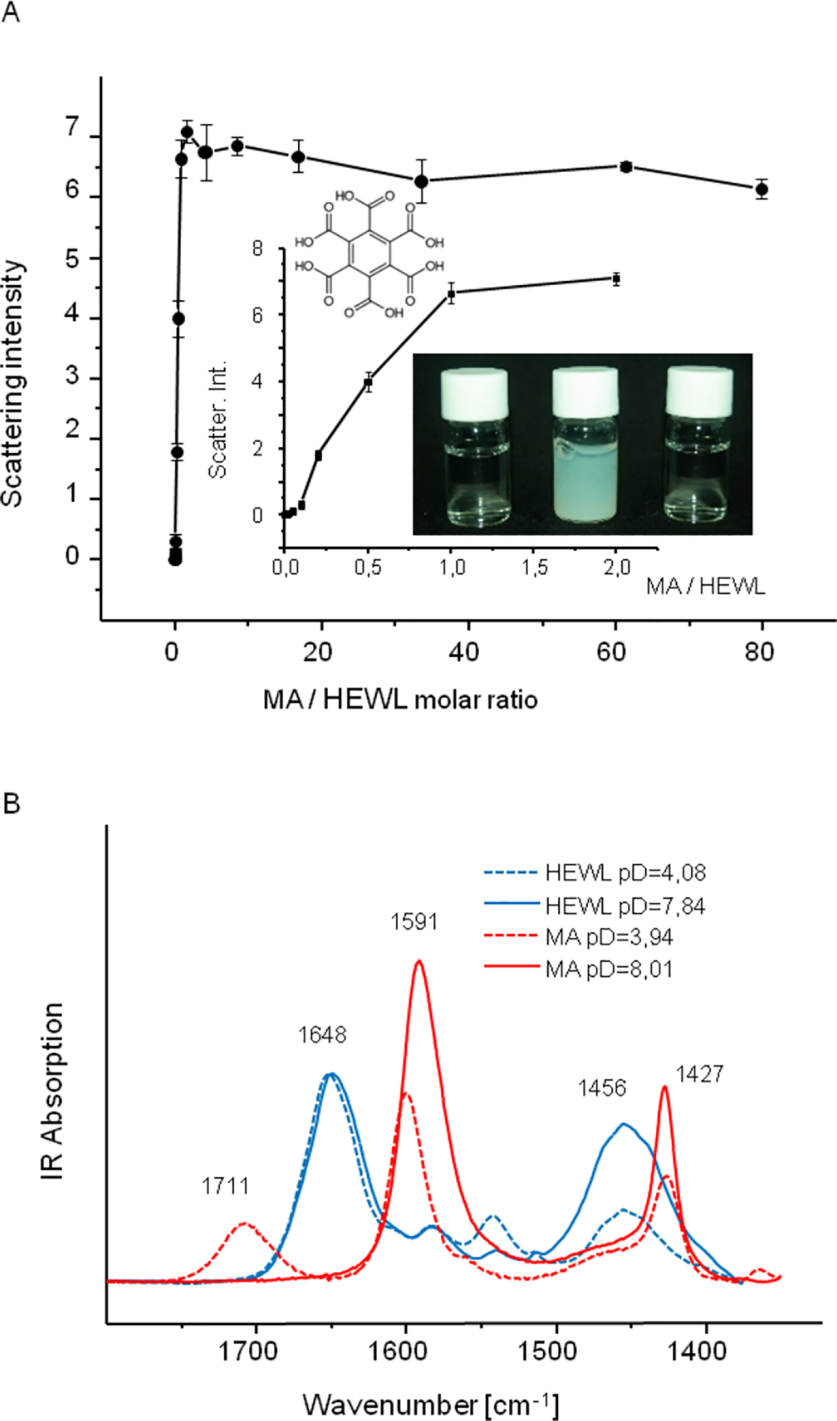
Co-precipitation of MA and HEWL. (A) Light scattering on HEWL-MA complexes formed at different molar ratios, HEWL wt. concentration was kept constant at 0,06%, pH was set at approx. 7,4; Inset shows magnification of the scattering dependency for the lowest MA:HEWL molar ratios; The photo shows 1 wt. %, pH 7,4 solutions of HEWL (left), MA (right) and their 1:1 mixture (middle); the molecular structure of MA is placed above the inset plot. (B) Infrared spectra of HEWL and MA dissolved in D_2_O at pD approx. 4 and 8 (solvent subtracted).

HEWL is a stable basic protein with the net electric charge of approximately +7 in the neutral environment [20], i.e. where MA dissociation equilibrium is shifted toward MA^5-^ and MA^6-^ anions [4, 5]. For the initial assessment of HEWL-MA electrostatic interactions, pH of 7.4–7.5 was selected to ensure, on the one hand, a possibly narrow distribution of mellitate anions with highest net negative charges and, on the other hand, to maintain both positive charge and structural stability of HEWL molecules. Fig 1A shows how mixing of such MA and HEWL solutions leads to immediate formation of insoluble electrostatic complexes which manifest by increased light scattering. It requires only a single mellitate ion per HEWL molecule to initiate formation of larger aggregates with a strong tendency to sediment. At approximately 3 mellitate anions per protein molecule, light scattering intensity reaches plateau and further increase of MA:HEWL ratio does not seem to either increase the signal or solubilize the pellet suggesting a rather defined stoichiometry of HEWL-MA complexes at least at this particular pH.

**Fig 2:**
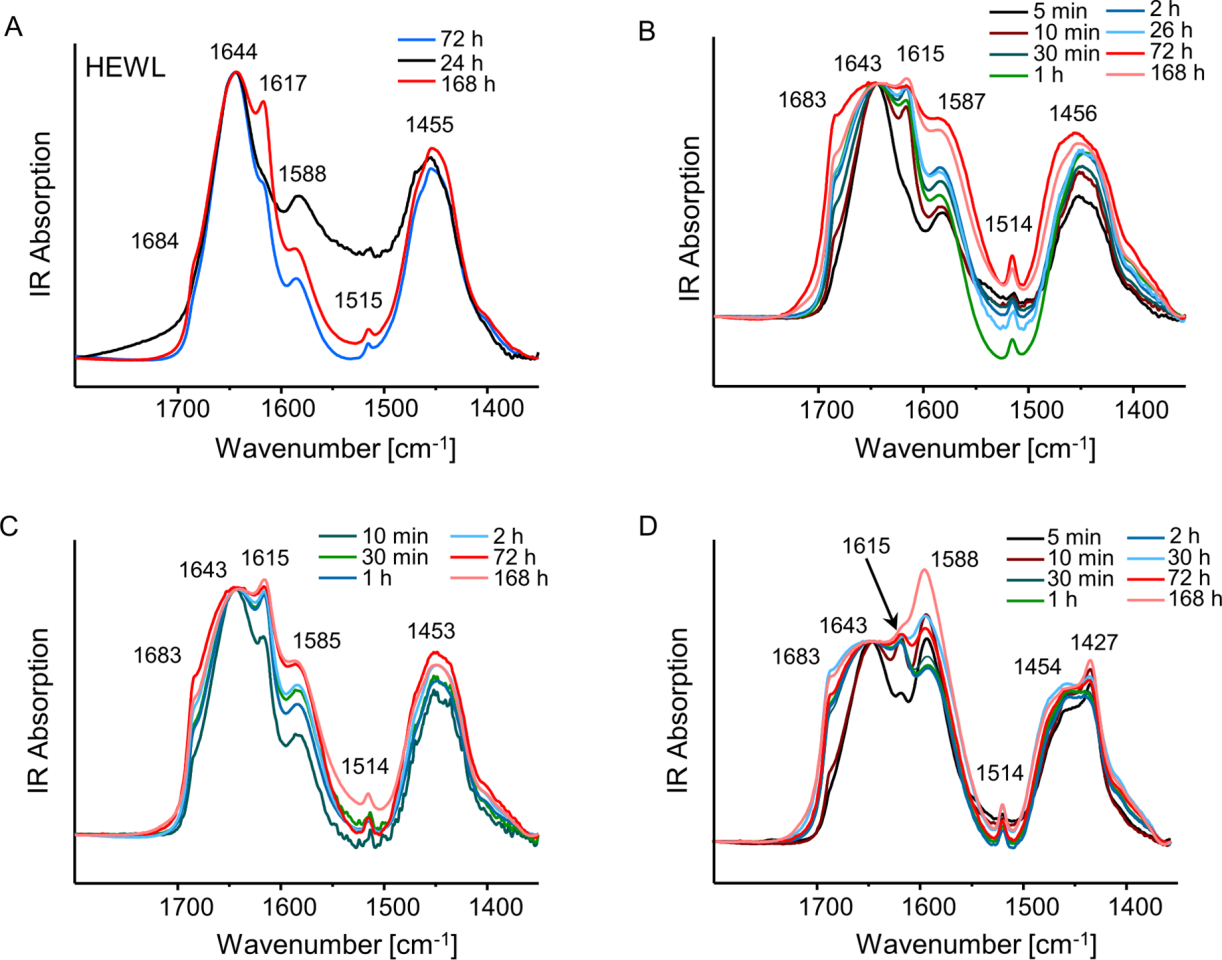
Time-dependent MA-induced misfolding of HEWL at 65°C probed by FT-IR spectroscopy. FT-IR spectra were acquired in the absence (A) and presence (B-D) of MA at different MA:HEWL molar ratios: 1:1 (B), 5,25:1 (C), and 21:1 (D). Concentration of HEWL in all samples was kept constant at 2 wt. %, pD of HEWL and MA solutions prior to mixing was adjusted to 7,5. The D_2_O-subtracted spectra correspond to pure precipitates (pellets) separated from the bulk sample on a centrifuge and re-suspended in D_2_O.

Compensation of surface electric charges on a globular protein could change (e.g. increase) the level of thermodynamic stability of the native state. We intended to verify this by subjecting HEWL-MA complexes (in comparison to HEWL in the absence of MA) to increasing temperature. As infrared absorption is less vulnerable than electronic circular dichroism (CD) to optical artefacts resulting from light scattering on insoluble particles we have selected FT-IR spectroscopy as an initial conformational probe of HEWL-MA complexes. While different mellitate species absorb near the conformation-sensitive amide I/I’ region of HEWL [26], they do not overlap this band in a manner preventing spectral analysis (Fig 1B), especially given the fact that these anions are effective in causing lysozyme precipitation already at very low concentrations. Fig 2 shows stacked FT-IR spectra of HEWL (A) and HEWL-mellitate (B-D) complexes formed at different molar ratios and subsequently subjected to prolonged incubation at 65^°^C. Because bending vibrations of H_2_O molecules would overlap this spectral region, all samples were prepared in D_2_O. The remarkable stability of native HEWL at neutral pD is reflected by the fact that the main component of amide I’ band at 1644 cm^-1^ (the wavenumber corresponding to the predominantly α-helical native structure of HEWL [27]) remains intact at least for the first 72 hours of incubation. However, in the spectrum collected after 1 week, a pair of peaks at 1617 cm^-1^ (strong) and 1684 cm^-1^ (very weak) attributed to exciton-split amide I’ component corresponding to the intermolecular β-sheets typically found in amorphous protein aggregates, becomes visible (Fig 2A). While we were expecting that this type of slow structural degradation of HEWL may be prevented in HEWL-MA complexes, the ensuing measurements proved this assumption to be wrong.

The pair of peaks hallmarking formation of non-native intermolecular β-sheets is slightly red-shifted (1615 cm^-1^ (strong) and 1683 cm^-1^ (weak)) in all HEWL aggregates formed in the presence of mellitate anions (Fig 2, panels B-D). More importantly, misfolding sets in very early during the incubation and even at the lowest MA concentration considered (when both HEWL and MA are at approximately 0.7 mM concentration–spectra in Fig 2B) the infrared features characteristic for the aggregated lysozyme are found within the first 10 minutes of incubation. When the MA:HEWL molar ratio is increased to 21:1 (Fig 2D), the intermolecular β-sheet forms already after 5 minutes and the transition appears to be complete within 1 hour. In the spectra corresponding to mature aggregates, the 1615 cm^-1^ band is somehow overlapped by 1588 cm^-1^ band originating from antisymmetric stretches of -COO¯ groups on both protein and mellitate anions.

**Fig 3:**
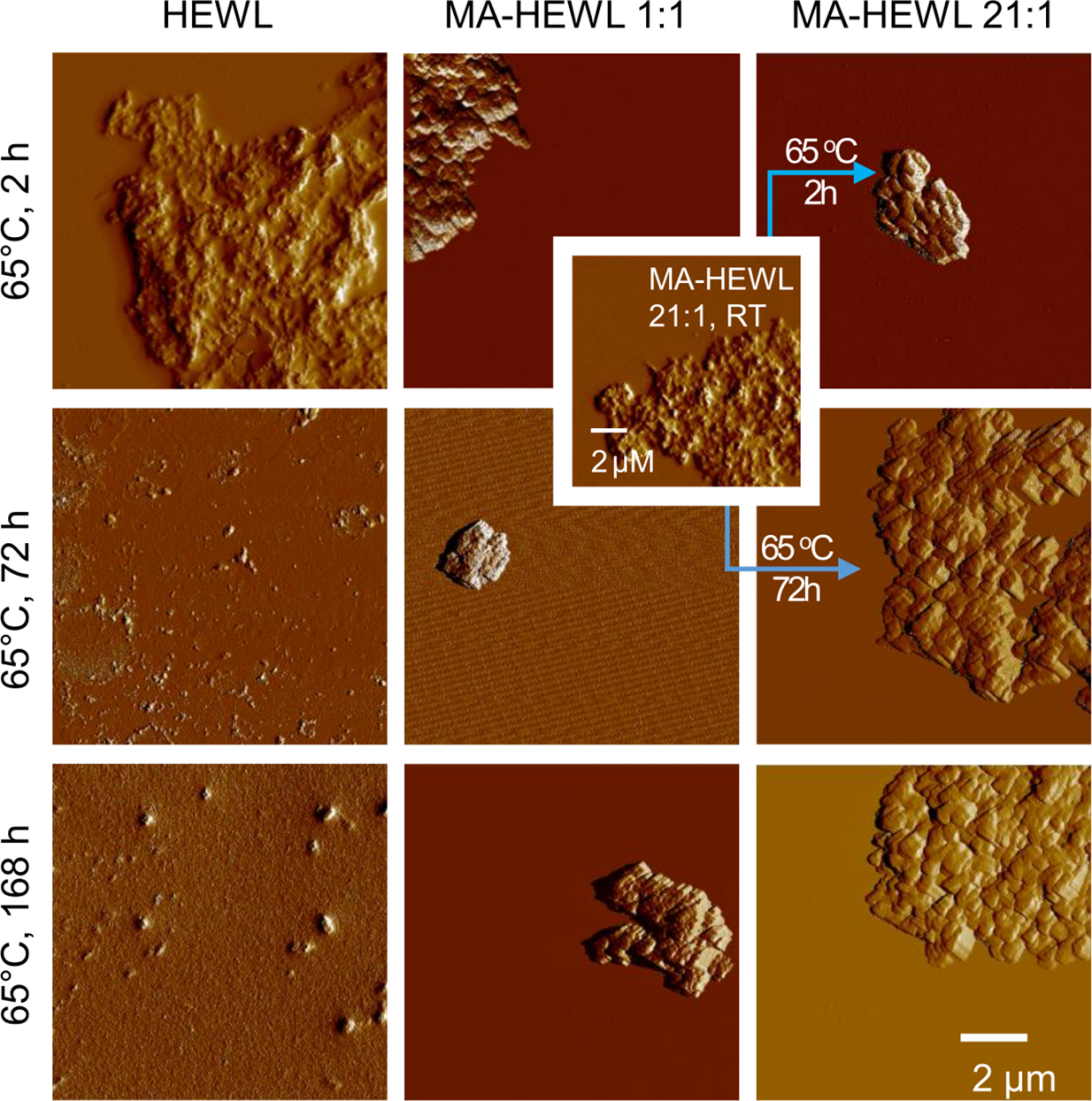
AFM images of representative specimen of HEWL and HEWL-MA aggregates obtained through prolonged incubation at 65°C. AFM images of representative specimen of HEWL aggregates obtained through prolonged incubation in the absence (left column) or presence (center and right columns) of MA under different conditions (2 wt. % HEWL-MA complexes in D_2_O under essentially the same conditions as used for the preparation of samples for the IR spectroscopic examination reported in Fig 2). The scale bar corresponds to all images. Inset image corresponds to fresh room temperature precipitate from 21:1 MA:HEWL sample before incubation at 65°C.

The time-lapse FT-IR spectra shown in Fig 2 reflect on a very powerful and unexpected effect of MA^5-^ and MA^6-^ anions which promote aggregation and misfolding of HEWL molecules. Tapping-mode AFM measurements were used to characterize morphologies of aggregates of misfolded HEWL formed under various conditions. The images shown in Fig 3 capture a significant difference between aggregates formed by HEWL in the absence and presence of mellitate anions.

Without MA, heating of HEWL results in small scattered aggregates with a diffuse appearance. Regardless of the length of incubation at 65 ^o^C, samples of HEWL aggregates are amorphous and heterogeneous in terms of size (left column in Fig 3). On the other hand, in the presence of MA, HEWL converts into densely-packed brick-like forms. There is no evidence of the small scattered aggregates characteristic for lysozyme undergoing aggregation in the absence of mellitate. The inset AFM image placed between the center and right columns corresponds to fresh, unheated precipitate from 21:1 MA:HEWL sample. Its diffuse morphology is clearly reminiscent of HEWL-only aggregates implying that the formation of brick-like structures requires temperature-induced unfolding of the protein.

**Fig 4:**
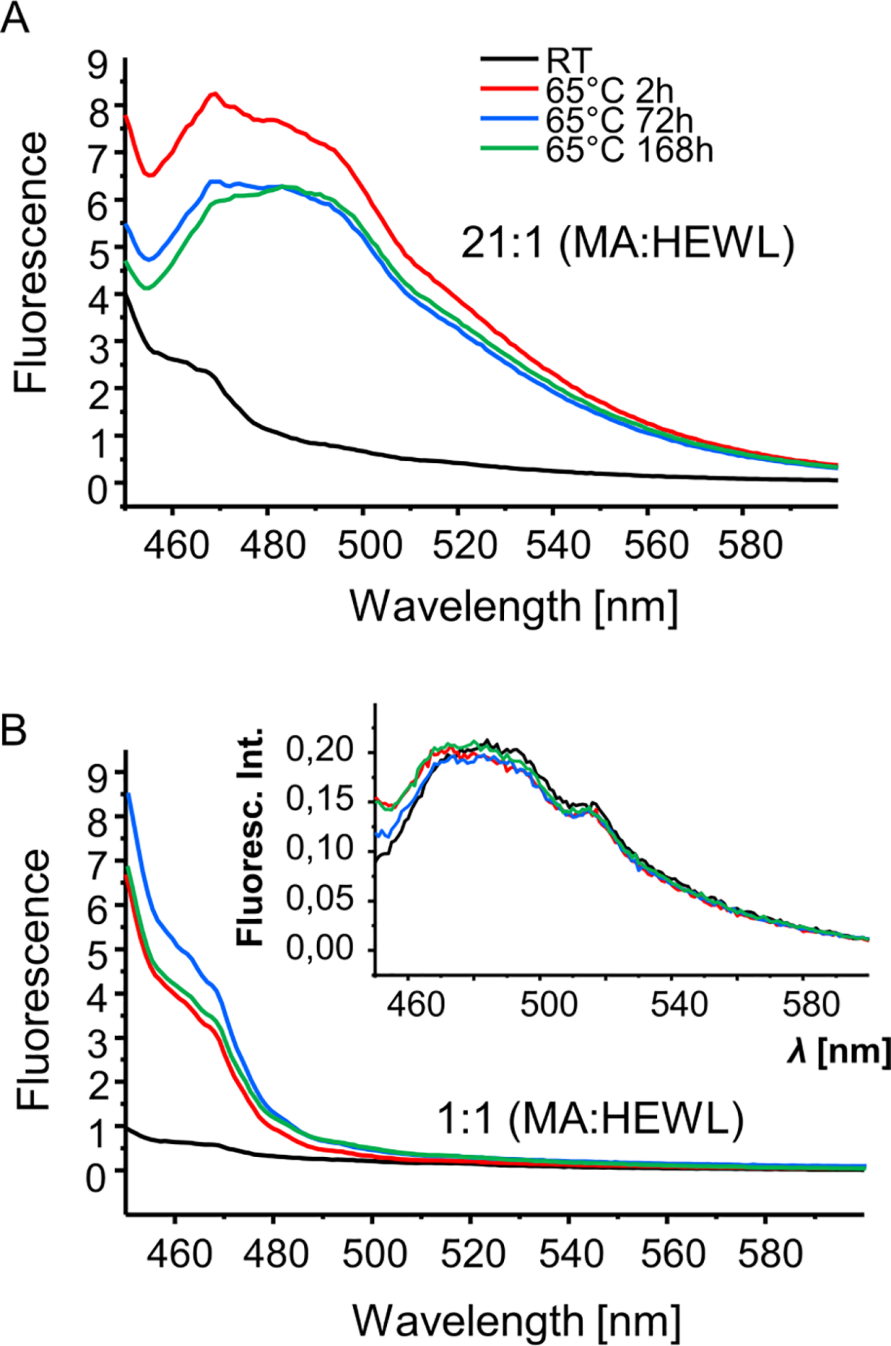
Fluorescence emission spectra of ThT-stained aggregates obtained through mixing of MA and HEWL solutions in D_2_O. Complexes were prepared by mixing samples at pD 7.6 and 21:1 (A) and 1:1 (B) MA:HEWL molar ratios. Prior to measurements, aggregates were incubated at 65^°^C / 300 rpm for specified periods of time (RT denotes spectra of freshly prepared samples). Inset in panel B shows emission spectra of ThT-stained HEWL samples in the absence of MA. All spectra shown were collected for identical ThT and protein concentrations, and using identical optical pathways and photomultiplier gain (voltage) settings; λ_exc._ = 440 nm.

Although no fibrillar amyloid-like entities were observed in these samples, the pronounced agglomeration could obscure an AFM-based detection of such structures. Hence, we used ThT: an amyloid-reporting fluorescent dye, to probe various aggregates [28–29]. In Fig 4, emission spectra of ThT added to HEWL-MA samples at different stages of incubation are presented. Clearly, even short 2h-long incubation at 65 ^o^C of 21:1 molar ratio complex causes a strong increase of fluorescence intensity at approx. 482 nm (Fig 4A). A slightly less pronounced effect is noted for the equimolar MA:HEWL sample, where the emission band is partly overlapped by the Rayleigh scattering of the broad excitation wavelength (centered around 440 nm). No similar increase of ThT fluorescence intensity is observed when the dye is added to HEWL aggregates formed after week-long incubation at 65 ^o^C without mellitate (inset in panel B). The ThT fluorescence data seems to suggest that the self-assembly of brick-like aggregates in HEWL-MA samples could be accompanied by formation of amyloid-like fibrils trapped in the matrix of less-ordered forms and therefore undetectable by AFM. Amorphous protein aggregates are expected to exhibit lower stability than fibrils (e.g. [30–31]). Such HEWL-MA aggregates are likely to trap ThT causing a moderate increase in quantum yield of its fluorescence following from a partial restriction of dye’s intramolecular rotational freedom which would be entirely unrelated to and independent of presence of amyloid fibrils. Such scenario is possible in light of the molecular-rotor-like photophysical behavior of ThT [32–33]. It is known that certain amorphous aggregates can enhance ThT emission to a limited degree [34]. Still, it cannot be entirely ruled out that HEWL-MA aggregates do contain tiny fractions of fibrils which cannot be detected by AFM due to trivial dilution.

**Fig 5:**
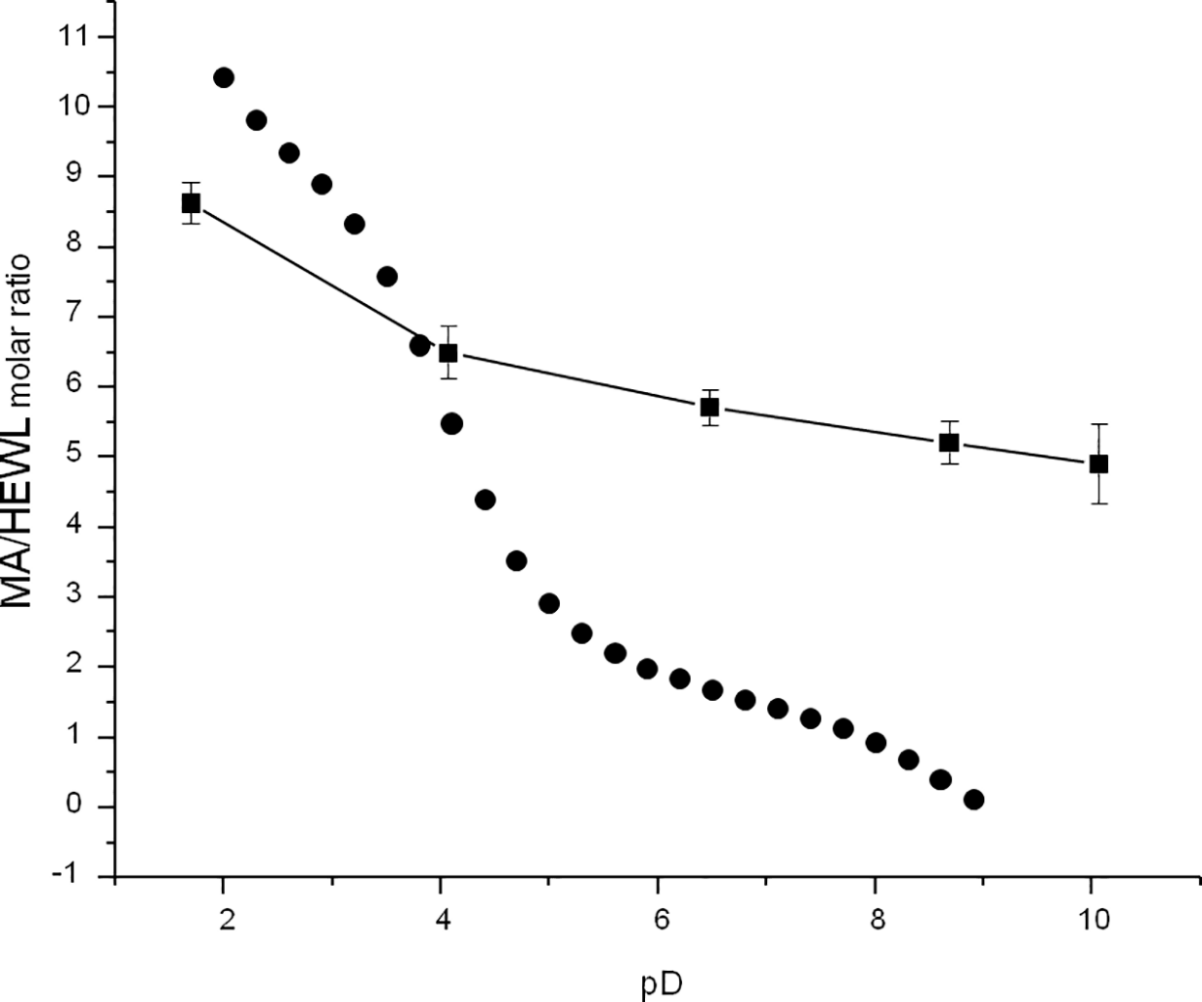
Dependency of HEWL-MA complex stoichiometry on pD. Approximate stoichiometries of HEWL-MA complexes precipitating from D_2_O-based mixtures of MA and HEWL (at the initial 21:1 molar ratio) at different pD (black squares connected with straight lines). Samples of freshly mixed MA and HEWL were subjected to 30 min incubation at 65°C and subsequently centrifuged; pellets were alkalized with a portion of diluted NaOD to pD = 12 resulting in solubilization of aggregates. Ratio of IR absorption at 1590 and 1650 cm^-1^ was used to estimate MA:HEWL molar ratio in the precipitate. The overlaid plot (black dots) corresponds to “theoretical” stoichiometry obtained as a molar ratio of MA and HEWL ions required to give neutral net charge of the complex in the absence of other ions (based on pH-dependent protonation equilibria of both HEWL and MA uncorrected for isotopic effects).

Strong electrostatic attraction between mellitate anions and HEWL cations is arguably the main driving force of the self-assembly. In the system where HEWL and MA^n-^ co-precipitate at sub-millimolar concentrations, the reminder of dissolved salt (Cl^-^ and Na^+^ are necessarily introduced with pD-adjusted stock solutions of HEWL and MA, respectively) is, at most, at sub-millimolar level and as such cannot significantly perturb the electrostatics of the self-assembly nor drive the association by enhancing hydrophobic effect. In fact, the removal of traces of salt from both fresh and temperature-annealed HEWL-MA aggregates through extensive dialysis against deionized water does not affect their constitution (S1 Fig, panel A). On the other hand, upon the addition of 0,2 M NaCl fresh precipitates dissolve immediately, although this treatment has a quite limited effect on HEWL-MA aggregates subjected to 1-hour-long incubation at 65 ^o^C (S1 Fig, panel B). This outcome is understandable in light of the fact that electrostatic interactions (which are most important for stabilization of fresh precipitates) are sensitive to high ionic strength whereas aged aggregates are additionally stabilized by hydrogen bonding of intermolecular β-sheets which are much less affected by ionic strength. Early forming precipitates in HEWL-MA samples trap mellitate anions, and the stoichiometry of protein-mellitate binding depends on pH (pD) of aggregation in a rather intuitive manner: the more acidic environment (the less negative charge on MA^n-^ anions) the higher is the number of mellitate anions required to compensate for increasingly positive charge on a lysozyme monomer (Fig 5). A naïve “theoretical” plot predicting stoichiometry of HEWL-MA complexes at various pH (pD) calculated for known pKa values of MA and simulated net electric charges of HEWL may only explain the general trend visible in the experimental data. There are several reasons for that; for example pKa values of protonable groups change upon formation of salt-bridges and subsequent burial in solvent-sequestered clefts.

**Fig 6:**
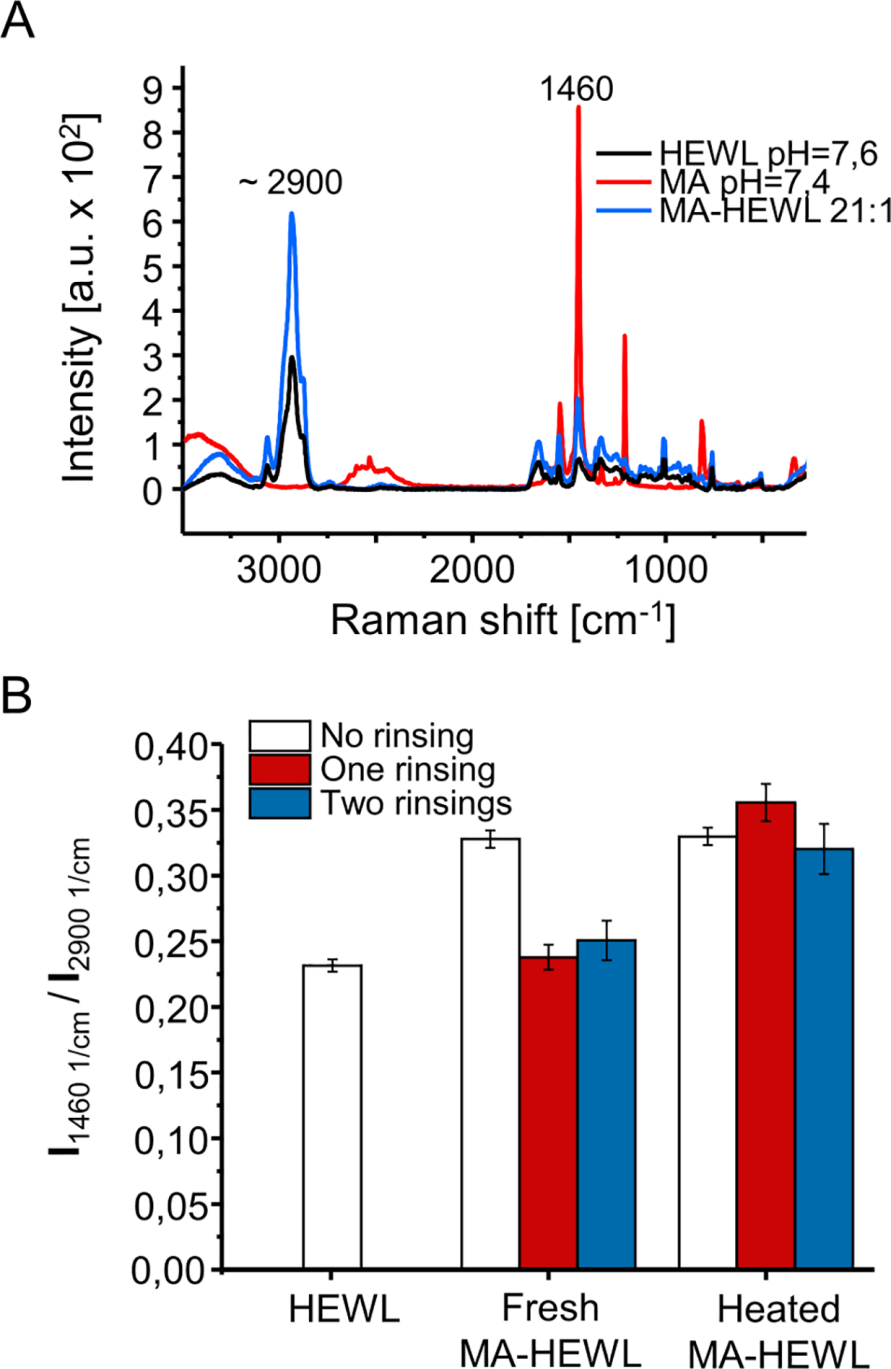
Raman spectroscopic detection of MA trapped within aggregated HEWL. (A) Raman spectra of HEWL, MA, and their mixture. (B) Ratio of Raman intensities at 1460 cm^-1^ and 2900 cm^-1^ of HEWL-MA complexes before and after rinsing with water and neat HEWL; values above 0.25 reflect the overlapping Raman band of trapped MA. Molar ratio MA:HEWL in the samples was 21:1. Incubation at 65°C was conducted for 20 minutes.

In the case of amyloidogenic self-assembly of certain proteins, pH conditions promoting aggregation-prone intermediate states often render dissolved protein highly charged which decelerates aggregation due to repulsive electrostatic interactions [35]. In such situations, presence of dissolved salts providing Debye screening of these unfavorable interactions strongly accelerates transition of singly dispersed monomers into aggregates. This is well exemplified by the case of insulin aggregation at low pH in the presence of NaCl (e.g. [35]). While chloride anions shield positively charged insulin monomers and dimers and enhance formation of insulin fibrils, they are not trapped within the growing amyloid [36]. The fibril is ultimately stabilized by non-ionic interactions. Through the removal of chloride anions, structural mismatches that could weaken these interactions are avoided (at the expense of limited electrostatic frustration). Thus, it was interesting to see whether MA^5-^ and MA^6-^ anions are trapped within the mature aggregates of HEWL, or, just like chloride ions in the case of insulin fibrillation, are ultimately pushed out of the proteinaceous aggregate. Because Raman spectra of mellitate anions feature a strong and narrow band at approximately 1460 cm^-1^ assigned to symmetric stretches of -COO¯ groups [37], we have used Raman spectroscopy to detect MA trapped within HEWL aggregates. Raman scattering at 1460 cm^-1^ stemming from protein’s ionized carboxyl groups is relatively low due to dilution, whereas, in contrast to proteins, none of mellitate species exhibits any Raman scattering at 2900 cm^-1^ due to the lack of C-H bonds (Fig 6A). Hence the ratio of Raman intensities at 1460 and 2900 cm^-1^ was used to quantify residual MA. The histograms in Fig 6B show how following rounds of elution of aggregates with water affect this ratio. For freshly precipitated aggregates from 21:1 MA:HEWL sample the ratio is clearly higher than for the neat protein, but even with a single round of rinsing with water (volume of rinsing water was twice the volume of the MA:HEWL sample) the intensity ratio drops back to the levels obtained for HEWL indicating depletion of MA. This outcome contrasts with the case of aggregates incubated at 65^°^C for 20 minutes, implying a firm entrapment of mellitate anions within the aggregate.

**Fig 7:**
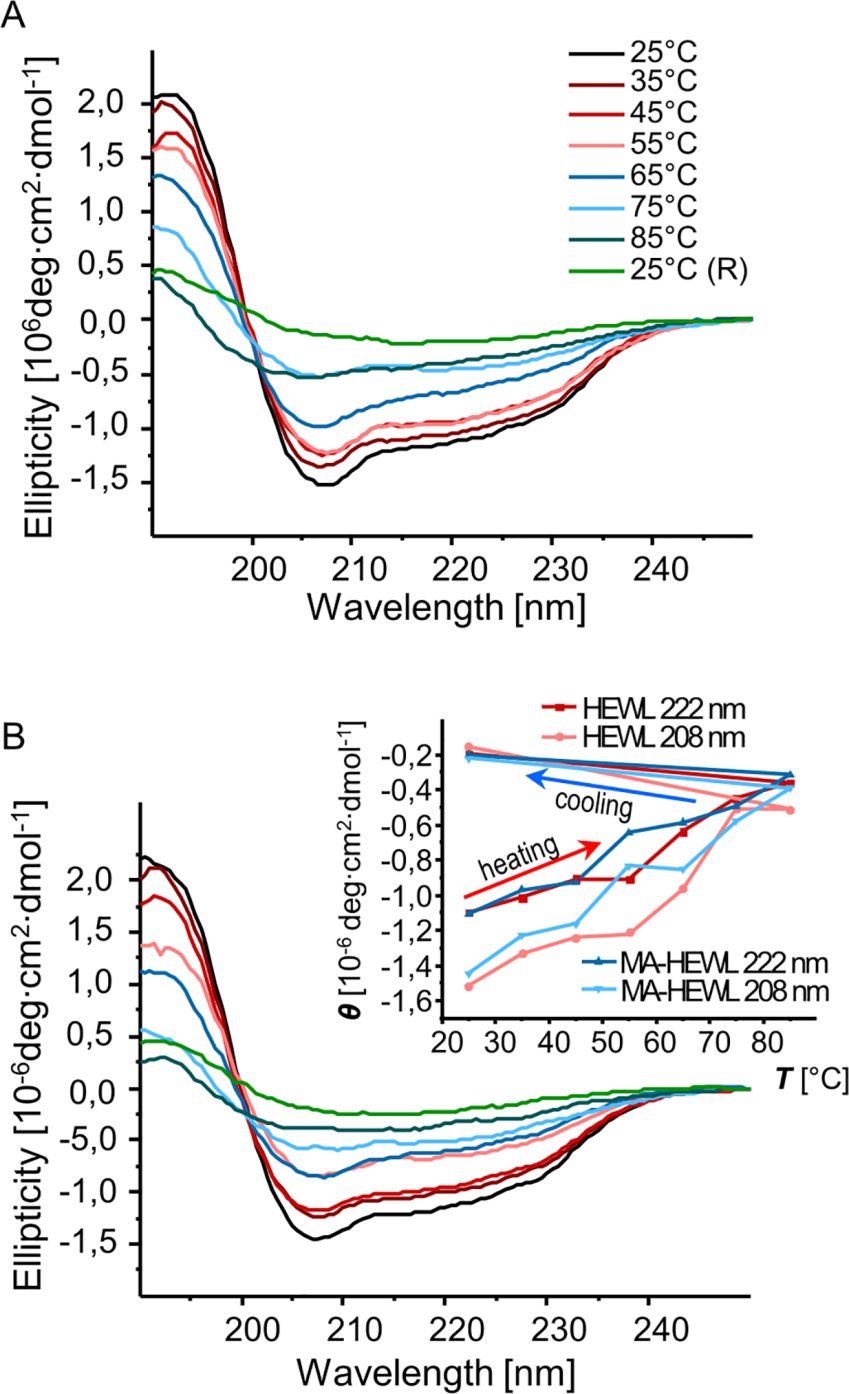
MA-induced aggregation of HEWL traced by CD spectroscopy. Far-UV CD spectra of HEWL (A), and of HEWL in the presence of MA at 1:1 molar ratio (B) at increasing temperatures and after cooling to 25 ^o^C, pD of samples was 7.4. Inset shows quantitative plots of ellipticity at 208 and 222 nm for either case.

There are several possible mechanisms through which mellitate anion may facilitate aggregation of HEWL. For example, direct protein-mellitate interactions may destabilize the native state of HEWL increasing the pool of partly unfolded aggregation-prone intermediates. Alternatively, the role of mellitate may be confined to overcoming barriers (of both entropic and enthalpic nature) of drawing several protein molecules in direct vicinity of each other through electrostatics-mediated sandwiching of MA^5-^/MA^6-^ between HEWL monomers. In order to address this uncertainty, we have carried out a comparative characterization of HEWL’s secondary structure in the presence and absence of mellitate anions (at the 1:1 MA:HEWL molar ratio) using far-UV CD (Fig 7). Samples for the measurements were prepared at low concentration typically required for far-UV CD and significantly below those used for FT-IR / AFM experiments. In this way, at least on the experimental time-scales used here, the freshly prepared HEWL-MA samples did not exhibit strong light scattering suggesting that local protein-anion interactions did not translate into cascade self-assembly leading to precipitation. This, in turn, allowed us to obtain high-quality far-UV CD spectra and focus on the interactions of singly dispersed HEWL molecules and mellitate anions. CD spectra in Fig 7 reveal a gradual denaturation of native lysozyme at increasing temperature. On the whole, the process consisting in slow flattening of helical signals at 208 and 222 (broader and poorly resolved) nm takes a similar course independent of whether MA was added to the sample. The apparent irreversibility of temperature-induced denaturation of HEWL is likewise unaffected by the presence of mellitate anions. However, the plotted dependencies of molar ellipticities of HEWL on temperature (inset in Fig 7B) reveal a sharper decrease of CD signals at 55 ^o^C in the presence of MA. Hence, while binding of mellitate to HEWL at ambient conditions does not appear to promote denaturation per se (the starting absolute ellipticity values at 25 ^o^C are very similar), it may make the protein more susceptible to subsequent thermal denaturation.

**Fig 8:**
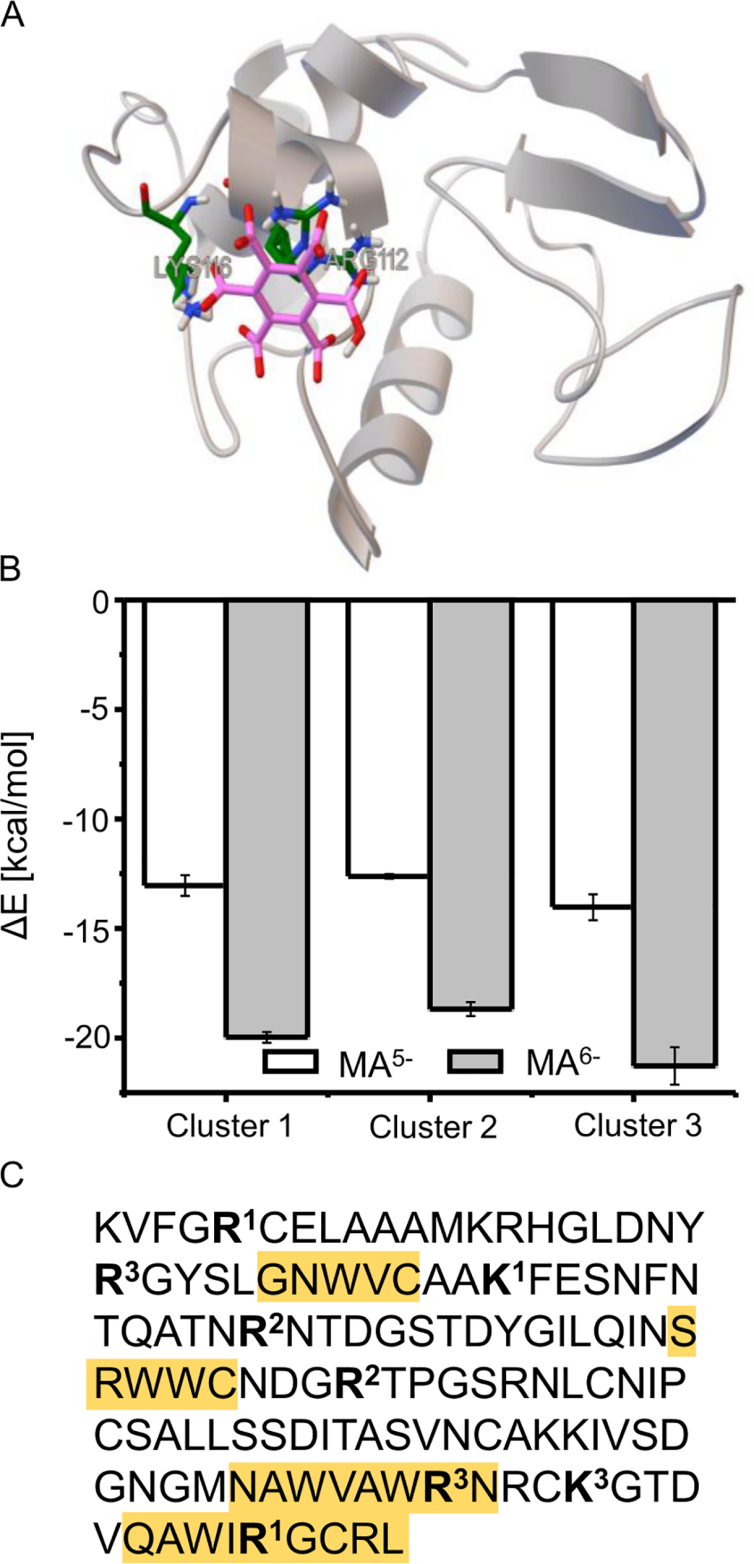
Hypothetical docking interactions between HEWL and MA^5-^ and MA^6-^ anions. (A) Visualization of docking MA^5-^ anion on HEWL native monomer involving residues Lys116 and Arg112 (according to AutoDock). (B) Binding energies of MA^5-^ and MA^6-^ to three different clusters of positively charges amino acid residues on the native HEWL molecule: Cluster 1: Arg5, Lys33, Arg1250; Cluster 2: Arg45, Arg68; Cluster 3: Arg21, Arg112, and Lys116. (C) Primary structure of HEWL with aggregation-prone segments (according to TANGO) marked in yellow and the MA^5-^ / MA^6-^ –binding clusters (residues) in bold (number in superscript corresponds to the cluster number).

In order to shed light on possible scenarios of HEWL-mellitate docking interactions, Autodock software was employed [22]. Fig 8A presents one of the typical results of docking simulation involving clusters of positively charged amino acid residues (here: Lys116 and Arg112) on the surface of HEWL and MA^5-^. The simulations yielded three cases of most energetically-favored docking modes (Fig 8B). The residues involved in these cases are: Arg5, Lys33, and Arg125 (Cluster1); Arg45, Arg68 (Cluster 2); Arg21, Arg112, and Lys116 (Cluster 3). In each case, replacing MA^5-^ with MA^6-^ strengthens the binding. Because there are at most only three positively charged residues binding a mellitate anion with twice larger negative charge this could provide a route for “dimerization” of HEWL monomers through a negatively-charged patch of a sandwiched MA^5-^ (MA^6-^) anion. In Fig 8C, the amino acid sequence of HEWL is displayed with the regions of mellitate-binding clusters marked in bold with the number in superscript corresponding to the cluster number, as defined above. Aggregation-prone regions of the HEWL sequence were selected according to TANGO–a statistical-mechanics-based algorithm [23–25] and were highlighted in yellow. We note, that some residues belonging to clusters 1 and, especially, cluster 3 lie within, or in proximity of, aggregation-prone regions placed close to the C-terminal part of HEWL sequence. While this approach offers only a very hypothetical idea of how MA^5-^/MA^6-^ ions may trigger aggregation of lysozyme, it does provide a potentially verifiable scenario of molecular events leading from the folded native state to amorphous aggregate. Such a scenario appears to resonate with the experimental data gathered in this work showing that presence of highly diluted tiny, but extremely charged anions can lead to misfolding of otherwise stable folded globular protein.

The fact that mellitate anions precipitate native HEWL through a direct binding and at sub-millimolar concentrations proves that this effect is different from those of the Hofmeister series [38]. It is well established that surface charged residues play an important role in maintaining protein stability [39] and an approach based on increasing the surface charges (“supercharging” proteins) has been successfully utilized in engineering of misfolding-resistant protein variants (e.g. [40]). As surface binding of MA^5-^ and MA^6-^ anions works exactly in the opposite direction, destabilization of the native structure observed for HEWL complexated by MA can be rationalized. Importantly, for HEWL in particular, the long-range of electrostatic interactions within the native state appear to play a key role in maintaining its stability [41–42], thus binding of a multivalent anion with high charge density could perturb the native balance of these interactions and result in populating partly disordered and possibly aggregation-prone states.

## Conclusions

Extremely charged mellitate anions strongly bind to chicken lysozyme under non-denaturating conditions and in the sub-millimolar concentration range causing its precipitation and facilitating misfolding and aggregation upon subsequent heating. The capacity of MA^5-^ and MA^6-^ anions to cause salting-out of soluble globular protein at such a low concentration through formation of a network of ionic interactions is very interesting, although so far practically unexplored. From the standpoint of biophysical studies on protein stability and misfolding, mellitate anions may provide possibly an interesting tool to access the aggregated state even under quasi-physiological conditions and without the need to introduce high concentrations of chemical denaturants or salts. As electrostatics play the key role in mellitate-protein interactions, it is interesting to see whether similar molecules could be used for therapeutic selective targeting of basic protein in vivo [43].

## Supporting information

S1 Fig Additional data on effects of ionic strength on MA-HEWL aggregates. (A) Light scattering at 532 nm on precipitates of MA-HEWL complexes formed at 21:1 molar ratio and subsequently heated for 1 h at 65 oC (red), or kept at ambient conditions (RT / blue) before (w/o) and after 30-min-long dialysis against 25-fold volume of deionized water. (B) Light scattering at 532 nm on precipitates of MA-HEWL complexes formed at 1:1 molar ratio and subsequently heated for 1 h at 65 oC (red), or kept at ambient conditions (RT / blue) before (w/o) and after addition of NaCl to its final concentration of 0,2 M. The results clearly indicate that presence of tiny residual concentrations of NaCl is not required for stabilization of fresh and heated MA-HEWL precipitates (A), and that high ionic strength has the capacity to dissolve fresh precipitates while heat-annealed aggregates withstand high NaCl concentration.
(DOCX)

## References

[1] Benner SA, Devine KG, Matveeva LN, Powell DH. The missing organic molecules on Mars. Proc Natl Acad Sci USA. 2000; 97(6):2425–2430. doi: 10.1073/pnas.040539497 10706606 10.1073/pnas.040539497 PMC15945

[2] Blanco Y, Rivas LA, Ruiz-Bermejo M, Parro V. Immunological detection of mellitic acid in the Atacama desert: Implication for organics detection on Mars. Icarus. 2013; 224(2):326–333.

[3] Stalport F, Coll P, Szopa C, Cottin H, Raulin F. Investigating the photostability of carboxylic acids exposed to Mars surface ultraviolet radiation conditions. Astrobiology. 2009; 9(6):543–549. doi: 10.1089/ast.2008.0300 19663761 10.1089/ast.2008.0300

[4] Apelblat A, Bešter-Rogač M, Barthel J, Neueder R. An analysis of electrical conductances of aqueous solutions of polybasic organic acids. Benzenehexacarboxylic (mellitic) acid and its neutral and acidic salts. J Phys Chem B. 2006; 110(17):8893–8906. doi: 10.1021/jp057371s 16640450 10.1021/jp057371s

[5] Kvaratskhelia E, Kvaratskhelia R. The electrolytic dissociation of mellitic acid. J Solution Chem. 2008; 37(8):1063–1070.

[6] Zhou T, Lucy CA. Separation of carboxylates by hydrophilic interaction liquid chromatography on titania. J Chromatogr A. 2010; 1217(1):82–88. doi: 10.1016/j.chroma.2009.11.016 19945708 10.1016/j.chroma.2009.11.016

[7] Inabe T. Mellitate anions: unique anionic component with supramolecular self-organizing properties. J Mater Chem. 2005; 15(13):1317–1328.

[8] Karle IL, Rajesh, YBRD, Ranganathan S. Crystal engineering: A unique cyclic assembly of a 40 membered module composed from two alternating units each of benzenehexacarboxylic acid (mellitic acid, MA) and 2, 5-bis-(4-pyridyl)-1, 3, 4-oxadiazole (4-BPO): Assembly of modules to macromolecules by intermolecular hydrogen bonding. J Chem Crystallogr. 2009; 39(3):201.

[9] Minemawari H, Naito T, Inabe T. Control of Molecular Packing in the ET Based Conductor by Supramolecular Mellitate Networks (ET = Bis (ethylenedithio) tetrathiafulvalene). Cryst Growth Des. 2009; 9(11):4830–4833.

[10] Karle IL, Rajesh YB, Ranganathan S. Conformational variability exhibited by mellitic acid anions (MA− n) with organic amine cations: Structures with an abundance of NH⋅ s OC hydrogen bonds and a paucity of hydrogen bonds. J Chem Crystallogr. 2005; 35(10):835–845.

[11] McMenimen KA, Hamilton DG. Mellitic Triimides: C_3_-Symmetric, Three-Electron Acceptors for Supramolecular Chemistry. J Am Chem Soc. 2001; 123(26):6453–6454. 11427087 10.1021/ja016043c

[12] Park LY, Hamilton DG, McGehee EA, McMenimen KA. Complementary C_3_-Symmetric Donor-Acceptor Components: Cocrystal Structure and Control of Mesophase Stability. J Am Chem Soc. 2003; 125(35):10586–10590. doi: 10.1021/ja036540o 12940741 10.1021/ja036540o

[13] Kiyotsukuri T, Takemoto N, Tsutsumi N, Sakai W, Nagata M. Regular network polyesters from benzenepolycarboxylic acids and glycol. Polymer. 1995; 36(26):5045–5049.

[14] Nagata M, Kiyotsukuri T, Moriya T, Tsutsumi N, Sakai W. Novel regular network polyimide films from mellitic acid and aliphatic and aromatic diamines or diisocyanates. Polymer. 1995; 36(13):2657–2662.

[15] Wu LP, Yamamoto M, Kuroda-Sowa T, Maekawa M, Fukui J, Munakata M. Crystal structure and magnetic properties of manganese (II) mellitate,[Mn_2_{C_6_(COO)_6_}(H_2_O)_6_][Mn (H_2_O)_6_]· 2H_2_O with two-dimensional layered structure and three-dimensional hydrogen bonding networks. Inorg Chim Acta. 1995; 239(1–2):165–169.

[16] Humphrey SM, Mole RA, Thompson RI, Wood PT. Mixed Alkali Metal/Transition Metal Coordination Polymers with the Mellitic Acid Hexaanion: 2-Dimensional Hexagonal Magnetic Nets. Inorg Chem. 2010; 49(7):3441–3448. doi: 10.1021/ic902527e 20205381 10.1021/ic902527e

[17] Mozhaev VV, Šikšnis VA, Melik‐Nubarov NS, Galkantaite NZ, Denis GJ, Butkus EP, Martinek K. Protein stabilization via hydrophilization. Eur J Biochem. 1988; 173(1):147–154. 2451606 10.1111/j.1432-1033.1988.tb13978.x

[18] Bucci E, Salahuddin A, Bonaventura J, Bonaventura C. Characterization of the ionizable groups interacting with anionic allosteric effectors of human hemoglobin. J Biol Chem. 1978; 253(3):821–827. 23382

[19] Mallette MF. A pH 7 buffer devoid of nitrogen, sulfur, and phosphorus for use in bacteriological systems. J Bacteriol. 1967; 94(2):283–290. 5341860 10.1128/jb.94.2.283-290.1967 PMC315037

[20] Colton IJ, Anderson JR, Gao J, Chapman RG, Isaacs L, Whitesides GM. Formation of protein charge ladders by acylation of amino groups on proteins. J. Amer. Chem. Soc. 1997; 119(52), 12701–12709.

[21] Plater JM, Harrison WTA, A geomimetic synthesis of mellite: a mineral containing a benzene ring, J Chem Res. 2015; 39(5):279–281.

[22] Morris GM, Huey R, Lindstrom W, Sanner MF, Belew RK, Goodsell DS, Olson AJ. Autodock4 and AutoDockTools4: automated docking with selective receptor flexiblity. *J Comput Chem*. 2009; 30(16):2785–91. doi: 10.1002/jcc.21256 19399780 10.1002/jcc.21256 PMC2760638

[23] Rousseau F, Schymkowitz J, Serrano L. Protein aggregation and amyloidosis: confusion of the kinds? Curr Opin Struct Biol. 2006; 16(1):118–126. doi: 10.1016/j.sbi.2006.01.011 16434184 10.1016/j.sbi.2006.01.011

[24] Fernandez-Escamilla AM, Rousseau F, Schymkowitz J, Serrano L. Prediction of sequence-dependent and mutational effects on the aggregation of peptides and proteins. Nat Biotechnol. 2004; 22(10):1302–1306. doi: 10.1038/nbt1012 15361882 10.1038/nbt1012

[25] Linding R, Schymkowitz J, Rousseau F, Diella F, Serrano L, A comparative study of the relationship between protein structure and beta-aggregation in globular and intrinsically disordered proteins. J Mol Biol. 2004; 342(1):345–353, doi: 10.1016/j.jmb.2004.06.088 15313629 10.1016/j.jmb.2004.06.088

[26] Johnson BB, Sjöberg S, Persson P. Surface complexation of mellitic acid to goethite: An attenuated total reflection Fourier transform infrared study. Langmuir. 2004; 20(3):823–828. 15773110 10.1021/la035471o

[27] Blake CCF, Koenig DF, Mair GA, North ACT, Phillips DC, Sarma VR. Structure of hen egg-white lysozyme: a three-dimensional Fourier synthesis at 2 Å resolution. Nature. 1965; 206(4986):757–761. 5891407 10.1038/206757a0

[28] Naiki H, Higuchi K, Hosokawa M, Takeda T. Fluorometric determination of amyloid fibrils in vitro using the fluorescent dye, thioflavine T. Anal Biochem. 1989; 177(2):244–249. 2729542 10.1016/0003-2697(89)90046-8

[29] LeVine H. Quantification of β-sheet amyloid fibril structures with thioflavin T. Methods Enzymol. 1999; 309:274–284. 10507030 10.1016/s0076-6879(99)09020-5

[30] Zurdo J, Guijarro JI, Dobson CM. Preparation and characterization of purified amyloid fibrils. J Am Chem Soc. 2001; 123(33):8141–8142. 11506581 10.1021/ja016229b

[31] Piejko M, Dec R, Babenko V, Hoang A, Szewczyk M, Mak P, Dzwolak W. Highly amyloidogenic two-chain peptide fragments are released upon partial digestion of insulin with pepsin. J Biol Chem. 2015; 290(10):5947–5958. doi: 10.1074/jbc.M114.608844 25586185 10.1074/jbc.M114.608844 PMC4358232

[32] Stsiapura VI, Maskevich AA, Kuzmitsky VA, Uversky VN, Kuznetsova IM, Turoverov KK. Thioflavin T as a molecular rotor: fluorescent properties of thioflavin T in solvents with different viscosity. J Phys Chem B. 2008; 112(49):15893–15902. doi: 10.1021/jp805822c 19367903 10.1021/jp805822c

[33] Amdursky N, Erez Y, Huppert D. Molecular rotors: what lies behind the high sensitivity of the thioflavin-T fluorescent marker. Acc Chem Res. 2012; 45(9):1548–1557. doi: 10.1021/ar300053p 22738376 10.1021/ar300053p

[34] Chang ESH, Liao TY, Lim TS, Fann W, Chen, RPY. A new amyloid-like ß-aggregate with amyloid characteristics, except fibril morphology. J. Mol. Biol. 2009; 385(4), 1257–1265. doi: 10.1016/j.jmb.2008.11.009 19041877 10.1016/j.jmb.2008.11.009

[35] Nielsen L, Khurana R, Coats A, Frokjaer S, Brange J, Vyas S, Fink AL. Effect of environmental factors on the kinetics of insulin fibril formation: elucidation of the molecular mechanism. Biochemistry. 2001; 40(20):6036–6046. 11352739 10.1021/bi002555c

[36] Babenko V, Surmacz-Chwedoruk W, Dzwolak W. On the function and fate of chloride ions in amyloidogenic self-assembly of insulin in an acidic environment: salt-induced condensation of fibrils. Langmuir. 2015; 31(7):2180–2186. doi: 10.1021/la5048694 25615018 10.1021/la5048694

[37] Jehlicka J, Edwards HGM, Villar SEJ. Raman spectroscopic study of mellite—a naturally occurring aluminium benzenehexacarboxylate from lignite—claystone series of the Tertiary age. Spectrochim Acta, Part A. 2006; 65(1):229–234. 10.1016/j.saa.2005.10.036 16503193

[38] Bye JW, Falconer RJ. Three stages of lysozyme thermal stabilization by high and medium charge density anions. J Phys Chem B. 2014; 118(16):4282–4286. doi: 10.1021/jp412140v 24684707 10.1021/jp412140v

[39] Strickler SS, Gribenko AV, Gribenko AV, Keiffer TR, Tomlinson J, Reihle T, Makhatadze GI. Protein stability and surface electrostatics: a charged relationship. Biochemistry. 2006; 45(9):2761–2766. doi: 10.1021/bi0600143 16503630 10.1021/bi0600143

[40] Lawrence MS, Phillips KJ, Liu DR. Supercharging proteins can impart unusual resilience. J Am Chem Soc. 2007; 129(33):10110–10112. doi: 10.1021/ja071641y 17665911 10.1021/ja071641y PMC2820565

[41] Halskau Ø, Perez-Jimenez R, Ibarra-Molero B, Underhaug J, Muñoz V, Martinez A, Sanchez-Ruiz JM. Large-scale modulation of thermodynamic protein folding barriers linked to electrostatics. Proc Natl Acad Sci USA. 2008; 105(25):8625–8630. doi: 10.1073/pnas.0709881105 18550823 10.1073/pnas.0709881105 PMC2438433

[42] Azia A, Levy Y. Nonnative electrostatic interactions can modulate protein folding: molecular dynamics with a grain of salt. J Mol Biol. 2009; 393(2):527–542. doi: 10.1016/j.jmb.2009.08.010 19683007 10.1016/j.jmb.2009.08.010

[43] Abdolvahabi A, Shi Y, Rhodes NR, Cook NP, Martí AA, Shaw BF. Arresting amyloid with coulomb’s law: acetylation of ALS-linked SOD1 by aspirin impedes aggregation. Biophys. J. 2015; 108(5): 1199–1212. doi: 10.1016/j.bpj.2015.01.014 25762331 10.1016/j.bpj.2015.01.014 PMC4375441

