# Supplementary material for: Mellitate: A multivalent anion with extreme charge density causes rapid aggregation and misfolding of wild type lysozyme at neutral pH
Source: PLoS One. 2017 Oct 30;12(10):e0187328. doi: 10.1371/journal.pone.0187328 (PMC5662172; doi:10.1371/journal.pone.0187328)
Supplement: S1 Fig — (A) Light scattering at 532 nm on precipitates of MA-HEWL complexes formed at 21:1 molar ratio and subsequently heated for 1 h at 65 oC (red), or kept at ambient conditions (RT / blue) before (w/o) and after 30-min-long dialysis against 25-fold volume of deionized water. (B) Light scattering at 532 nm on precipitates of MA-HEWL complexes formed at 1:1 molar ratio and subsequently heated for 1 h at 65 oC (red), or kept at ambient conditions (RT / blue) before (w/o) and after addition of NaCl to its final concentration of 0,2 M. The results clearly indicate that presence of tiny residual concentrations of NaCl is not required for stabilization of fresh and heated MA-HEWL precipitates (A), and that high ionic strength has the capacity to dissolve fresh precipitates while heat-annealed aggregates withstand high NaCl concentration. (DOCX) [file pone.0187328.s001.docx]

Figure S1

**Mellitate: a multivalent anion with extreme charge density causes rapid aggregation and misfolding of wild type lysozyme at neutral pH**

*Grzegorz Ścibisz, Robert Dec, and Wojciech Dzwolak*

Faculty of Chemistry, Biological and Chemical Research Centre, University of Warsaw, Pasteur 1 St., 02-093 Warsaw, Poland

**Figure S1.** (A) Light scattering at 532 nm on precipitates of MA-HEWL complexes formed at 21:1 molar ratio and subsequently heated for 1 h at 65 ^o^C (red), or kept at ambient conditions (RT / blue) before (w/o) and after 30-min-long dialysis against 25-fold volume of deionized water. (B) Light scattering at 532 nm on precipitates of MA-HEWL complexes formed at 1:1 molar ratio and subsequently heated for 1 h at 65 ^o^C (red), or kept at ambient conditions (RT / blue) before (w/o) and after addition of NaCl to its final concentration of 0,2 M.

The results clearly indicate that presence of tiny residual concentrations of NaCl is not required for stabilization of fresh and heated MA-HEWL precipitates (A), and that high ionic strength has the capacity to dissolve fresh precipitates while heat-annealed aggregates withstand high NaCl concentration.
